# Studies on the Uncrosslinked Fraction of PLA/PBAT Blends Modified by Electron Radiation

**DOI:** 10.3390/ma13051068

**Published:** 2020-02-28

**Authors:** Rafał Malinowski, Krzysztof Moraczewski, Aneta Raszkowska-Kaczor

**Affiliations:** 1 Łukasiewicz Research Network—Institute for Engineering of Polymer Materials and Dyes, 87100 Toruń, Poland; aneta.raszkowska-kaczor@impib.pl; 2 Institute of Materials Engineering, Kazimierz Wielki University, 85064 Bydgoszcz, Poland; kmm@ukw.edu.pl

**Keywords:** polylactide, biodegradable blends, irradiation, crosslinking, degradation

## Abstract

The results of studies on the uncrosslinked fraction of blends of polylactide and poly(butylene adipate-*co*-terephthalate) (PLA/PBAT) are presented. The blends were crosslinked by using the electron radiation and triallyl isocyanurate (TAIC) at a concentration of 3 wt %. Two kinds of samples to be investigated were prepared: one contained 80 wt % PLA and the other contained 80 wt % PBAT. Both blends were irradiated with the doses of 10, 40, or 90 kGy. The uncrosslinked fraction was separated from the crosslinked one. When dried, they were subjected to quantitative analysis, Fourier transform infrared spectroscopy (FTIR) measurements, an analysis of variations in the average molecular weight, and the determination of thermal properties. It was found that the electron radiation caused various effects in the studied samples, which depended on the magnitude of the radiation dose and the weight fractions of the components of the particular blends. This was evidenced by the occurrence of the uncrosslinked fractions of different amounts, a different molecular weight distribution, and the different thermal properties of the samples. It was also concluded that the observed effects were caused by the fact that the processes of crosslinking and degradation took place mostly in PLA, while PBAT appeared to be less susceptible to the influence of the electron radiation.

## 1. Introduction

Studies on the effects of electron radiation on the properties of conventional polymers are already being carried out and have been for many years [1]. The development of knowledge in this field as well as learning the new phenomena and consequences of the interaction of this radiation with polymer materials resulted in an increase in the number of novel applications of polymers and put the use of the industrial radiation treatment into a new perspective. Changes occurring in the molecular structure of the polymers are the most common effects of the treatment with electron radiation. They are caused by the formation of various kinds of ions and radicals in the materials being radiation modified. This, in turn, leads to the occurrence of many processes, including synthesis, grafting, crosslinking, or radiation degradation [2]. As a result of these processes and due to specified conditions of the radiation treatment, novel polymer materials of modified properties and new potential applications are being produced.

Issues associated with the radiation treatment of polymers are complex, which results from the fact that various processes of different intensity may simultaneously occur in the polymers being modified this way. Therefore, the final effects of the irradiation, i.e., mostly crosslinking or degradation at different degrees, depend, e.g., on the yield of the individual processes [3]. Additionally, an active site (usually a radical one) being formed due to the irradiation, may move along a polymer chain. Thus, the processes induced by the absorption of the electron radiation energy by a polymer may occur in places of chain different with relation to location of the active site created originally. This results partially from the chemical structure of the macromolecules and partially from the conditions of the radiation treatment, mainly the radiation dose magnitude, dose rate, and the irradiation atmosphere. For example, polyethylene (PE) easily undergoes radiation crosslinking, whereas polypropylene (PP) solely undergoes radiation degradation, during which the molecular weight decreases [4]. This is caused by the presence in PP of lateral methyl groups that favor oxidation of the polymer and, thus, its degradation.

Various effects of the radiation treatment also occur in the biodegradable polymers, such as polylactide (PLA), poly(ε-caprolactone) (PCL), or poly(butylene adipate-*co*-terephthalate) (PBAT) [5,6,7,8,9]. They depend, for example, on the structure of macromolecules, the orderliness of carbon atoms in the main chain or the presence of aromatic groups. They are, however, much less recognized, which may result from the anxiety associated with a possible decrease in the susceptibility to the biodegradability of these polymers due to crosslinking. Nevertheless, not all the biodegradable polymers undergo crosslinking because of treatment with the electron radiation only. For example, PLA [10,11,12], when treated with the electron radiation, undergoes, first of all, degradation [13,14,15]. On the contrary, in PCL [16,17,18] or in PBAT [19,20,21,22], the radiation degradation processes generally are not dominant [23,24,25].

The influence of the electron radiation on the properties of blends of the biodegradable polymers (like PLA/PCL, PLA/PBAT or PHBV/PLA) [26,27,28] is even less recognized or totally unexplained. This statement does not apply to radiation sterilization processes that generally use low radiation doses that do not affect the properties and structure of polymers. In the case of using higher doses (above 10 kGy), the effects of the irradiation are much more complex. Such a situation results from different interactions of particular components of the blends with the radiation [29,30,31,32,33,34]. The low miscibility or immiscibility of the components are additional factors affecting complexity of the issue [35]. In order to increase the miscibility of the polymers, it is recommended to add to such blends low-molecular weight compounds that increase crosslinking and improve interfacial adhesion [31,36,37,38,39,40,41]. Triallyl isocyanurate (TAIC) is one such compound, which is being applied most often.

Knowledge about the influence of the electron radiation on polymers and polymer blends, including the effects associated with the crosslinking, may be acquired as a result of the investigation of a crosslinked fraction and phenomena occurring within that fraction. On the other hand, the results of the examination of the fraction that remains uncrosslinked in spite of irradiation are almost totally unknown. Most often, the amount of it is less than that of other fractions, which does not, however, mean that this fraction has to be degraded. Getting knowledge about its properties when irradiated may be an excellent supplement to the results of investigation of the crosslinked fraction and may also be a kind of reflection of the latter. In addition, this knowledge may give some information on the crosslinking of the studied materials. It may also contribute to the elucidation of the issues that have not been fully explained earlier. Therefore, the authors of the present article undertook studies aimed at determination of changes in some properties of the uncrosslinked fraction of the two kinds of blends of biodegradable polymers, occurring when the electron radiation doses of different magnitudes were applied. The studies presented in this work are a continuation of our previous research on the radiation treatment of PLA, PBAT, and its blends [13,25,32]. Therefore, the blends of PLA/PBAT type were chosen for studies.

## 2. Materials and Methods

### 2.1. Materials

Polylactide (PLA), type 2003D (NatureWorks^®^, Minnetonka, MN, USA), with a melt flow rate (MFR) equal to 2.8 g/10min (2.16 kg, 190 °C), a density of 1.24 g/cm^3^, a melting temperature (T_m_) of 155 °C, a number-average molecular weight of ca. 91 kDa, and a weight-average molecular weight of ca. 166 kDa, has been used in this work. This polymer contained 3.5% of D monomer units. The second polymer was poly(butylene adipate-*co*-terephthalate) (PBAT), type FBlend C1200 (BASF, Ludwigshafen, Germany) with a melt flow rate (MFR) equal to 10 g/10 min (2.16 kg, 190 °C), a density of 1.25 g/cm^3^, a melting temperature (T_m_) of 115 °C, a number-average molecular weight of ca. 35 kDa, and a weight-average molecular weight of ca. 73 kDa. Moreover, triallyl isocyanurate (TAIC) with a density equal to 1.16 g/cm^3^ and a melting point of ca. 23–27 °C from Sigma-Aldrich GmbH (Munich, Germany) has also been applied. This compound was used in a liquid state as an agent to promote a crosslinking of the polymers upon the electron radiation. Dichloromethane (CH_2_Cl_2_) from Avantor Performance Materials Poland S.A. (Gliwice, Poland) was used during dissolution of the non-irradiated and irradiated samples in investigations of the uncrosslinked fraction, as well as in examination by gel permeation chromatography (GPC). Nitrogen (N_2_) type Premier (Air Products, Warsaw, Poland) was used in the differential scanning calorimeter (DSC) examination.

### 2.2. Apparatus

The co-rotating twin screw extruder type BTSK 20/40D (Bühler, Braunschweig, Germany), equipped with the screws of a 40 L/D ratio and 20 mm diameter, and the three-opening die head was intended to be prepared from granulated samples of the PLA/PBAT blends. A linear accelerator of electrons, type Elektronika 10/10, was used in the irradiation of the obtained samples of the granulates. Attenuated total reflectance Fourier transform infrared (FTIR-ATR) spectrometer, type Cary 630 (Agilent Technologies, Santa Clara, CA, USA), was meant for the examination of the changes in the macromolecules’ structure. In addition, gel permeation chromatograph (GPC), equipped with the set of two PLgel 5 µm MIXED-C columns, was designed for the investigation of the average molecular weights of the radiation modified samples. The last device applied in this work was a DSC, type DSC 1 STARe System (Mettler Toledo, Greifensee, Switzerland), designed for the determination of some thermal effects occurring in samples modified by electron radiation.

### 2.3. Sample Preparation

Samples of the uncrosslinked fractions were prepared in three stages. In the first stage, two kinds of granulated polymer blends of the PLA/PBAT type were prepared by using a co-rotating twin screw extruder type BTSK 20/40D (Bühler, Braunschweig, Germany) and a standard granulator. One kind of granule was the blend with the predominant content (80 wt %) of PLA, and the other one was the blend with the predominant content (80 wt %) of PBAT. TAIC in the amount of 3 wt % was added to each blend while being extruded. The extrusion was carried out at the temperatures of the particular barrel zones (I, II, III, and IV) equal to 180, 183, 186, and 190 °C, respectively, and at the die-head temperature of 190 °C [32]. Free degassing of possible gaseous products when released was applied at the screws with a length of L/D = 33. The screws rotational speed was constant (250 rpm). Before extrusion, the PLA was dried at 70 °C for 24 h, in order to avoid hydrolytic degradation. PBAT was not dried. The same temperature profile to extrude the two blends has been applied, because these were the lowest processing temperatures of PLA, which was present in both blends.

In the second stage, the prepared granulated blends were subjected to the radiation treatment with the use of a high-energy electron beam derived from accelerator type Elektronika 10/10 for the crosslinking of the studied materials. The applied radiation doses were 10, 40, or 90 kGy, the single dose not being larger than 20 kGy. This limitation was due to an increase in the temperature of the irradiated material, which might cause unwanted changes in the structure of the blends if larger doses were used. Therefore, some samples were irradiated several times. These doses were chosen because at the 10 kGy dose the crosslinking process began, at the 40 kGy dose the largest amount of gel fraction was obtained, and at the 90 kGy dose the degradation process was dominant. During the irradiation procedure, all the granulated samples were put on the belt conveyor moving at the speed of 0.3–1.2 m/min. The actual speed was related to the radiation dose absorbed by a polymer material being modified. The layer thickness of the irradiated samples was not larger than 20 mm. This ensured penetration of the irradiation beam on the all granules. Samples irradiation was carried out in the air.

In the third stage, the radiation crosslinked samples of the granulated blends were subjected to dissolution in CH_2_Cl_2_ in order to separate the uncrosslinked fraction from the crosslinked fraction. This solvent was chosen because the studied samples dissolve well in it. The non-irradiated samples (L0 and B0, as indicated in Table 1) were also subjected to dissolution in that solvent. The samples were dissolving at room temperature for 24 h. The obtained solutions were filtered through medium flow quantitative filter papers. The crosslinked fractions that remained on the filter papers were used to determine the contents of the gel fractions. The uncrosslinked fractions present in the filtrates were used to prepare samples meant for the basic examinations. For this purpose, the filtrates were put on glass Petri dishes and left until the solvent had freely evaporated. The obtained specimens of the uncrosslinked fractions, designated with the symbols as shown in Table 1, were investigated by using techniques such as Fourier transform infrared spectroscopy (FTIR), gel permeation chromatography (GPC), and differential scanning calorimetry (DSC).

### 2.4. Methodology of Research

The content of the uncrosslinked fraction (N_g_) of the studied samples was determined by a solvent extraction method, using CH_2_Cl_2_ as the solvent. All the specimens were subjected to dissolution at 20 ± 3 °C for 24 h. The mass ratios of the specimens and solvent were chosen so as to achieve a solution concentration (C_p_) of 2%. The obtained solutions were filtered through medium flow quantitative filter papers. The gel fraction, which remained on the filter papers, was dried at 50 °C for 24 h. Using the mass (W_o_) of a sample before it was subjected to dissolution and the mass (W_g_) of that sample after it was subjected to dissolution and dried (W_g_ referred to the mass of the gel fraction), the content of the uncrosslinked fraction (N_g_) in the particular samples was calculated according to the following relationship:

$$N_{g}=\left(1-\frac{W_{g}}{W_{o}}\right)\times 100\%$$

The attenuated total reflectance Fourier transform infrared (ATR-FTIR) spectra of the studied samples were recorded in transmittance mode at a constant spectral resolution of 4 cm^–1^, for the wavenumber ranged from 3700 to 500 cm^−1^, after acquiring 8 scans.

The changes in the number-average molecular weight (M_n_) and weight-average molecular weight (M_w_) of all samples was examined by gel permeation chromatography (GPC) conducted in CH_2_Cl_2_ at room temperature with an eluent flow rate of 0.8 mL/min, using a set of two PLgel 5 µm MIXED-C columns. About 2 mg of each sample was applied as part of the preparation of the solution that was to be injected in the GPC columns. Polystyrene standards were used. GPC results have been shown as curves of the peak intensity (I) vs. the eluent volume (V).

DSC measurements were performed under nitrogen with rate flow of 50 ml/min. About 3 mg of the sample was placed on an aluminum pan for sampling. The samples were successively: heated from 20 to 180 °C at 10 °C/min, annealed at 180 °C for 3 min, cooled to 15 °C at 10 °C/min, and reheated to 180 °C at a rate of 10 °C/min. The second heating cycle was used in the analysis of the thermal properties of the studied samples. The glass transition temperature (T_g_), cold crystallization temperature (T_cc_), melting temperature (T_m_), cold crystallization enthalpy (H_cc_), and melting enthalpy (H_m_) were determined.

## 3. Results

### 3.1. Quantitative Determination of the Uncrosslinked Fraction

**Figure 1:**
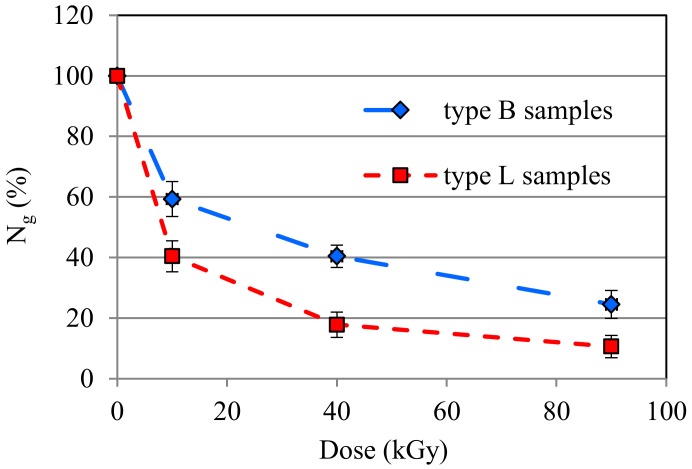
Results of the content of the uncrosslinked fraction (N_g_) of the samples type L and B.

Dependences of the content of the uncrosslinked fraction (N_g_) of the samples L and B on the magnitude of the radiation dose are shown in Figure 1. As can be seen, the N_g_ values of the B samples are on the average a dozen or so percent larger than those of the L samples. This indicates a higher susceptibility of PLA to the radiation crosslinking in the presence of TAIC than that of PBAT. Larger N_g_ values, corresponding to smaller contents of the gel fraction of PBAT, result from the chemical structure of the macromolecules of that polymer, mostly from the presence of aromatic groupings that enable the absorption of a part of the electron radiation and the dissipation of it in the form of heat. This is connected to the so-called protective effect and to making at least some of the active sites, which may move along polymer chains, inactive [42,43]. Such a situation is the reason for the creation of a smaller number of crosslinking bonds and, thus, for the formation of a smaller amount of the gel fraction while, at the same time, a larger amount of the uncrosslinked fraction is formed.

From Figure 1, it also follows that the N_g_ values of the samples of both types rapidly decrease as the radiation dose increases. Already, the smallest radiation dose causes a reduction in the N_g_ values down to ca. 40% (sample L10) and 59% (sample B10). The largest dose, in turn, causes a decrease in the values of N_g_ to ca. 10% (sample L90) and 25% (sample B90). Considering the values of the confidence intervals of the particular results, it has to be stated that essential variations in the values of Ng occurred for all irradiated samples except for samples L40 and L90. A reduction in the contents of the uncrosslinked fraction in the particular samples upon the increase in the radiation dose points out the fact that the crosslinking becomes more effective. However, the radiation modified blends do not undergo the complete crosslinking since the N_g_ values do not fall down to zero. This may indicate that the applied amount of TAIC could be insufficient for the complete crosslinking of the studied blends. Besides, the amount of an uncrosslinked fraction at the level of 10% or 25% is relatively large anyway.

The data shown in Figure 1 also indicate that the gel fraction does not form in the non-irradiated samples when solely TAIC was used. This is proved by the maximum N_g_ values of these samples, which are associated with the easy dissolution of those materials in the applied solvent. It is an important piece of information because not all the low molecular weight multifunctional compounds show a similar effect [44]. Some of them, e.g., trimethylopropane triacrylate (TMPTA), may cause the formation of small amounts of the gel fraction in the case of several polymers already at the stage of processing these materials and without the application of the electron radiation.

### 3.2. FTIR Spectroscopy

**Figure 2:**
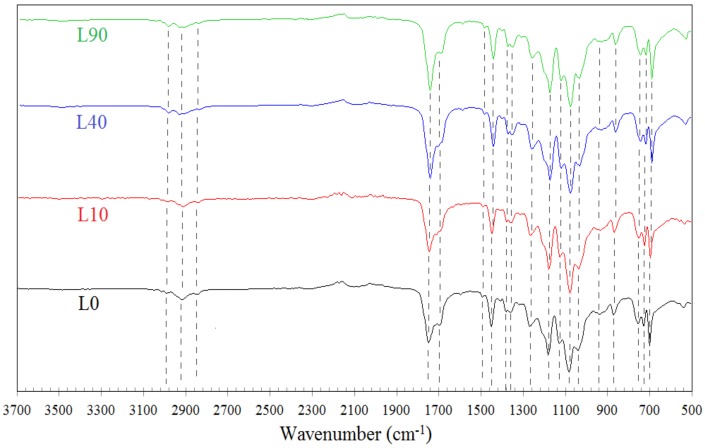
Results of the Fourier transform infrared spectroscopy (FTIR) measurements of the samples type L.

**Figure 3:**
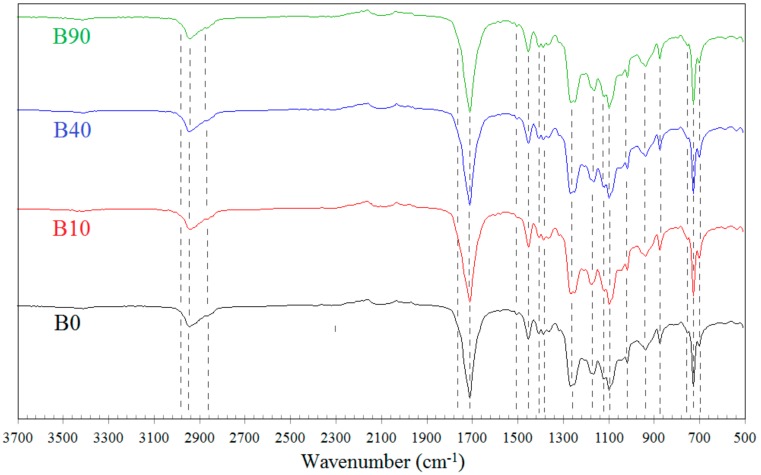
Results of the FTIR measurements of the samples type B.

The uncrosslinked fractions of the studied samples may exhibit various properties, which may result from different macromolecular structures of PLA and/or PBAT after irradiation. In addition, the blends of both types when irradiated may show slightly different weight percentages of PLA and PBAT compared to the original blends produced at the stage of extrusion, which may result from the crosslinking of both polymers at different degrees. This is proved to some extent by the N_g_ data. In the extreme case, the uncrosslinked fraction might be composed of solely one polymer if the other one would be completely crosslinked and adhesion at the interface between these two polymers would be insufficient. The FTIR spectra of the L type samples (L0, L10, L40, and L90) and those of the B type samples (B0, B10, B40, and B90) are shown in Figure 2 and Figure 3, respectively. The results of the FTIR measurements enable us to determine compositions of the studied samples.

Considering the FTIR spectra, only the bands characteristic of PLA or PBAT were taken into account [45,46,47]. In the case of PLA, there were analyzed bands assigned to (i) the asymmetric and symmetric stretching vibrations of the CH_3_ group present in the saturated hydrocarbons (2994 and 2946 cm^−1^, respectively), (ii) the stretching vibrations of the C=O group (1748 cm^−1^), (iii) asymmetric bending vibrations of the CH_3_ group (1452 cm^−1^), (iv) the deformation and symmetric bending vibrations of the CH group (1382 and 1360 cm^−1^, respectively), and (v) the stretching vibrations of the C–O–C group (1180, 1079, and 1042 cm^−1^) because C–O can form a bond with different atoms and groups, so the vibration absorptions are more complex. Moreover, the band at 869 cm^−1^ can be assigned to the amorphous phase and the one at 757 cm^−1^ to the crystalline phase of PLA [48]. However, other authors report that the band at 869 cm^−1^ can be assigned to the absorption of the (O–CH–CH_3_) ester and the one at 757 cm^−1^ can be assigned to the rocking vibration absorption of α-methyl [49]. The bond at 697 cm^−1^ is assigned to the vibrations of the carbonyl group (C=O). In the case of PBAT, in turn, the bands ascribed to (i) the asymmetric stretching vibrations of the CH_2_ group (2948 cm^−1^), (ii) the stretching vibrations of the C=O group of the ester bond (1711 cm^−1^), (iii) the skeletal vibrations of the aromatic ring (1504 cm^−1^), (iv) the in-plane bending vibrations of the CH_2_ group (1409 cm^−1^), (v) the symmetric stretching vibrations of the C–O group (1267 cm^−1^), (vi) the left–right symmetric stretching vibration absorption of the C–O group (1104 cm^−1^), (vii) the vibrations of the hydrogen atom of the aromatic ring (1018 cm^−1^), (viii) the symmetric stretching vibration of the trans C–O group (935 cm^−1^), and (ix) the bending vibration absorption of CH-plane of the benzene ring (727 cm ^−1^) were taken into account.

The bands characteristic of both PLA and PBAT appear in each spectrum, which indicates that all the studied samples contain macromolecules of the two polymers. The bands of the L and B samples that have been irradiated using the same radiation dose differ from one another in intensity only, which results from the predominant content of one of the polymers. It is also important that the positions of the characteristic bands in the spectra corresponding to the irradiated samples did not change, or changed only slightly, in respect of those relating to the non-irradiated samples. This points out that the studied samples contained no crosslinked macromolecules of both polymers.

### 3.3. Molecular Weight

**Figure 4:**
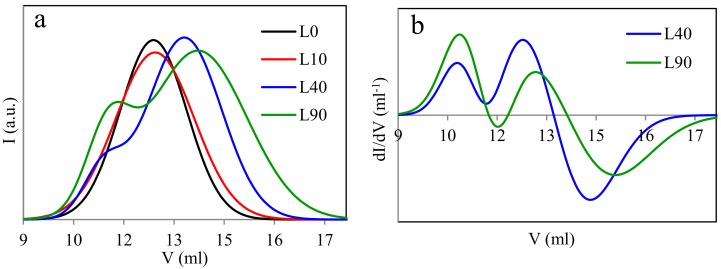
Results of the gel permeation chromatography (GPC) measurements for the samples with the predominant content of polylactide (PLA) (**a**—GPC curves for the samples type L; **b**—derivatives (*dI/dV*) of the GPC curves for the samples L40 and L90).

**Figure 5:**
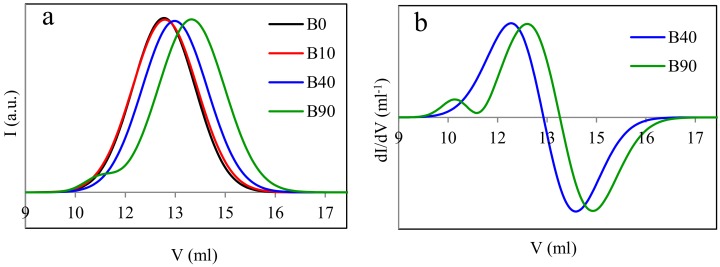
Results of GPC measurements for the samples with the predominant content of poly(butylene adipate-*co*-terephthalate) (PBAT) (**a**—GPC curves for the samples type B; **b**—derivatives (*dI/dV*) of the GPC curves for the samples B40 and B90).

Treatment with the electron radiation essentially influences the average molecular weights of the polymers being modified. This is connected with the degradation of the polymers, the change in the structure of macromolecules (e.g., branching and lengthening), or crosslinking (partial or complete). GPC data, illustrated in Figure 4 and Figure 5 and summarized in Table 2, indicate that effects occurring in the samples with the predominant content of PLA are somewhat different in comparison to those in the samples with the prevailing content of PBAT.

Two types of molecular weight distributions, i.e., monomodal and bimodal, were found in the case of the L samples (Figure 4). The non-irradiated blend and that irradiated with the dose of 10 kGy exhibit the monomodal distribution and molecular weights close to one another. These samples differ in the degree of polydispersion only, which is slightly higher in the irradiated sample. The distribution peak of the latter sample is lower and its full width at half maximum is larger compared to the non-irradiated sample. The higher degree of the polydispersion of the irradiated sample results from the partial radiation degradation of polylactide, which easily degrades when exposed to the electron radiation. The difference in the degree of polydispersion may also be caused by the fact that the discussed sample contains PLA (being susceptible to the radiation degradation) in the predominant amount. The monomodal distribution of the molecular weight of the non-irradiated sample (L0) is due to the fact that macromolecules of both polymers (PLA and PBAT) may probably have different hydrodynamic volumes. This, in turn, may result in the retention times of the macromolecules of different lengths being similar. Thus, despite the different molecular weights of the individual components of the blend, the molecular weight distribution of L0 sample can be a monomodal.

The bimodal distribution occurs in the sample irradiated with the dose of 40 kGy, which is more obvious when the derivative of the GPC curve is taken into account. Such a distribution is even more evident in the case of the sample irradiated with the dose of 90 kGy. The occurrence of the bimodal distribution indicates that the short macromolecules (degraded or non-degraded oligomeric) as well as the macromolecules of the molecular weight larger than that of the macromolecules of the non-irradiated sample are present in the samples L40 and L90. This may result from the formation of branched, elongated, or partially crosslinked structures. Furthermore, it can also be seen in Figure 4 that a shift in the positions of particular peaks in the direction of larger or smaller eluent volumes (V) appears as the radiation dose increases. The former direction corresponds to the samples containing macromolecules of the diminishing molecular weight, whereas the latter one corresponds to the samples L40 and L90 only, in which the molecular weight of a part of the macromolecules increases as the radiation dose rises. From Table 2, it also follows that the number average molecular weight of one of the fractions of the L samples (the fraction that contains macromolecules with smaller molecular weights, peak 2) decreased by a factor of almost six when the maximum radiation dose was applied. On the other hand, if the fraction containing macromolecules with larger molecular weights is concerned (peak 1), the larger molecular weight of sample L40, as compared to that of sample L90, may result from the more effective combining of some PLA macromolecules into more complex structures than in the sample irradiated with the dose of 90 kGy, which may facilitate the degradation of the PLA macromolecules.

The bimodal distribution does not practically occur in the B samples (Figure 5). It can only slightly be seen in the case of the sample irradiated with the 90 kGy dose. An insignificant peak of the position clearly shifted to the left in respect of the remaining peaks, which is more distinct on the derivative of the GPC curve, indicates a contribution to that sample of the macromolecules with a larger molecular weight. However, these macromolecules should not be completely crosslinked, but at most be elongated or partially crosslinked only. The monomodal distribution of the molecular weight of the B0 sample before radiation treatment is due to the same reason as for the non-irradiated L0 sample.

The shift in the positions of the highest peaks towards larger eluent volumes proves the decrease in the average molecular weights of the B samples, occurring with the increasing radiation dose. It can be due to the shortening of the macromolecules (degradation) or the reduction in the number of the longer macromolecules that undergo the crosslinking while forming the gel fraction. Thus, only the shorter macromolecules would remain in the studied sample, which had not undergone crosslinking nor degradation. Besides, while considering the results of the N_g_ determination (Figure 1), one can state that the number of the shorter macromolecules in the B samples is relatively high. The data summarized in Table 2 also point out that the number average molecular weights of the B samples decrease by the factor of three when they get irradiated with the maximum dose (peak 2). Thus, the reduction in the molecular weights of the B samples is smaller by the factor of two in relation to that of the L samples. This proves again that PLA is more susceptible to the radiation degradation compared to PBAT.

When considering the results of determination of both the content of the uncrosslinked fraction (N_g_) and the molecular weight, one may state that the occurrence of the bimodal distribution is closely dependent on both the weight fraction of PLA and the radiation dose magnitude. This distribution becomes more evident when the PLA weight fraction and the radiation dose increase. This may indicate that properties of the studied samples will be determined by the PLA phase. On the other hand, the PLA fraction of the B samples is relatively small. Thus, the properties of those samples should mainly be determined by the radiation dose magnitude, which was confirmed by the results of the DSC measurements (Section 3.4, Figure 7).

### 3.4. DSC Results

**Figure 6:**
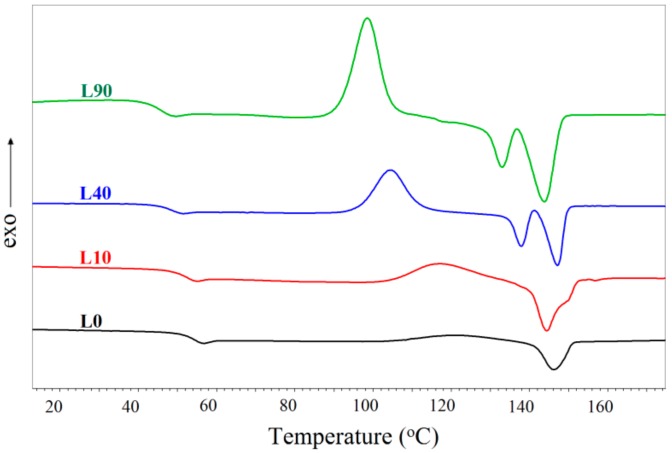
DSC curves recorded for the samples type L.

**Figure 7:**
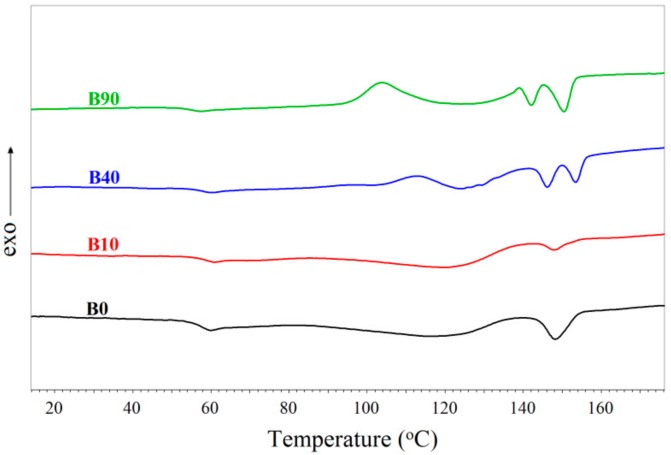
DSC curves recorded for the samples type B.

The DSC second heating curves recorded for the samples L and B are shown in Figure 6 and Figure 7, respectively. The data concerning particular phase transitions are summarized in Table 3. They include the glass transition temperature (T_g_) of the PLA phase, the cold crystallization temperature (T_cc_) of the PLA phase, the cold crystallization enthalpy (H_cc_) of the PLA phase, the melting temperature of the crystalline phases of PBAT (T_m_ (PBAT)) and PLA (T_m_ (PLA)), and the enthalpies of the melting of the crystalline phases of PBAT (H_m_ (PBAT)) and PLA (H_m_ (PLA)). Detailed discussions on the observed phenomena is presented for the samples L and B separately.

#### 3.4.1. Analysis of the DSC Results for Samples L

The lowering of the glass transition temperature by ca. 9 °C occurred in the region of the glass transition of PLA as the radiation dose was increased (Figure 6). This could be caused by the presence in the studied samples of the short macromolecules or oligomeric structures, acting as plasticizer and being, e.g., products of the radiation degradation of PLA or PBAT. The glass transition temperature of PBAT could not be determined since it lies in the region far below 0 °C (beyond the studied range).

The cold crystallization occurs in all the studied samples over the temperature range of ca. 102–125 °C. It appears only in the temperature region characteristic of the cold crystallization of PLA, which indicates that the polylactide phase of the samples was initially amorphous. A lack of the peak corresponding to the cold crystallization of PBAT indicates that the phase of this polymer exhibited maximum crystallinity. Besides, the PBAT fraction in the L samples was small and the effect connected with the cold crystallization of that polymer could hardly be noticed. From Figure 6, it also follows that the cold crystallization temperature of the PLA phase decreases and the cold crystallization enthalpy of that phase increases as the radiation dose rises. Such a relationship points out that the rate of ordering of the macromolecules increases as the radiation dose increases. This can occur when the macromolecules become shorter or adhesion at the interface improves (in the case of the polymer blends) and the transport barrier to crystallization becomes reduced. The latter effect seems to be confirmed by the reduction of the full width at half the maximum of the cold crystallization peaks with the rising radiation dose. The increase in the cold crystallization of PLA upon the rising radiation dose may also be caused by the nucleation effect of the shorter macromolecules of PBAT [50].

Slight changes in crystallinity of the studied samples could be observed as well. The crystallinity was determined from both the cold crystallization enthalpies and the enthalpies of melting of the crystalline phase. The obtained data indicated that the L0 and L10 samples were amorphous because the values of the enthalpies of cold crystallization and the melting of these samples were close to each other. The L40 and L90 samples already exhibited some crystallinity that increased as the radiation dose rose. This might confirm the conclusion that more extensive and quicker ordering of the macromolecules occurs in the samples irradiated with the maximum doses, i.e., these samples exhibit a greater ability to crystallize.

An analysis of the melting peaks of the crystalline phase gives information on the particularly interesting effects. Figure 6 illustrates melting of only the polylactide phase. The peak assigned to the melting of the PBAT phase cannot be seen because they occur in the region of the cold crystallization of PLA. The weight fraction of polylactide predominates in the discussed samples and the cold crystallization heat of PLA may be larger than the heat of the melting of the PBAT crystalline phase. The peaks ascribed to the melting of the PLA crystalline phase are single or double. The single peak concerns the non-irradiated sample (L0) only. In the case of the irradiated samples, the phase transition may be reflected by a single peak with two apexes or two peaks that are almost entirely separated, which can especially be seen in the curves of the L40 and L90 samples.

A single peak with two apexes, assigned to the melting of the crystalline phase, can often be observed in the case of PLA and its blends with other polymers [50,51,52]. It reflects the melting and recrystallization of original crystalline structures into more stable forms (in this case the melting peak occurs at lower temperatures) and the melting of the more stable crystalline structures (the melting peak occurs at higher temperatures). From Figure 6, it follows that the recrystallization into the more stable crystalline structures occurs more easily in the samples irradiated with larger doses. This is indicated by the decreasing ratio of the melting enthalpy corresponding to the lower temperature peak to that relating to the higher temperature peak.

In the case of two separated peaks that correspond to two different phase transitions, another effect can also occur. It concerns melting of two crystalline phases with different molecular weights, which can appear in the radiation modified samples. This would confirm the GPC results indicating that a bimodal distribution of molecular weights occurs in the samples irradiated with the largest doses. Two clearly separated peaks on the DSC curves, ascribed to the melting of the crystalline phases, correspond to the bimodal distribution of molecular weights in the same samples (irradiated with the doses of 40 or 90 kGy), which was observed on the GPC curves. Two melting peaks can also be seen in the case of the sample irradiated with the 10 kGy dose. However, one of them (occurring at a higher range of temperatures) is very small and the relevant bimodal distribution was not observed on the GPC curves. Nevertheless, the presence of this tiny peak is closely connected with a much larger degree of polydispersion of the L10 sample with respect to the remaining samples (Table 2). This fact may indicate that already the smallest radiation dose induces essential radiation processes in the studied sample. However, the discussed effect, which is associated with the melting of the crystalline phases of two fractions with different molecular weights, is less probable or does not predominate. This conclusion may be justified by the fact that only one peak assigned to the cold crystallization occurs on the DSC curves of all samples, which points rather out the melting of the crystalline phase and its further recrystallization.

The DSC results also show that the melting temperature of the crystalline phase, corresponding to both peaks, decreases as the radiation dose increases. This is especially visible when these peaks occur in the range of lower temperatures: the melting temperature drops from ca. 149 (sample L0) to ca 136 °C (sample L90). This fact is associated with a notable reduction in the molecular weight of the relevant samples, which can be confirmed by the GPC results. In the range of higher temperatures, the melting temperature of the crystalline phase of the L40 sample is by ca. 3 °C higher than that of the L90 sample, which results from a larger molecular weight of one of the fractions of the L40 sample as compared to the analogous fraction of the L90 sample (Table 2).

#### 3.4.2. Analysis of the DSC Results for Samples B

Several thermal effects of the phase transitions, occurring in the B samples (Figure 7), somewhat differ from those observed in the samples with the predominating PLA fraction. The direction of the change in the glass transition temperature upon the increasing radiation dose is similar to that occurring in the L samples. However, the reduction in the values of this temperature in the B samples irradiated with the maximum dose is smaller than that of the L samples and is equal to ca. 3 °C. This is due to the effect connected with the plasticization of the samples by a part of the degraded macromolecules of PLA or PBAT. However, the reduction in the glass transition temperature of the B samples, due to the plasticization of those materials by the degraded macromolecules of PLA or PBAT, is smaller compared to the L samples because the number average molecular weight of the degraded macromolecules in the B samples is larger than that in the relevant L samples, which was shown above (GPC results, Table 2). Thus, the ability of the plasticizer to reduce the glass transition temperature of the relevant samples decreases as the molecular weight of the plasticizer increases.

From Figure 7, it also follows that the DSC curves of the samples non-irradiated or irradiated with the 10-kGy dose exhibit the presence of two peaks corresponding to the melting of the crystalline phases of PBAT (at ca. 120 °C) and PLA (at ca. 150 °C). These curves do not include the cold crystallization peaks, contrary to the DSC curves of the L samples, in which the peaks assigned to the cold crystallization of PLA were observed. A lack of the peak of the cold crystallization of PBAT indicates that this phase already exhibited some crystallinity. On the other hand, the peak of the cold crystallization of PLA is invisible because this peak and the peak corresponding to the melting of the crystalline phase of PBAT occur over the same temperature range and one may assume that the thermal effect of the melting of the crystalline phase of PBAT is larger than that of the cold crystallization of PLA. The larger thermal effect of the melting of PBAT might result from the fact that the PBAT phase was initially crystalline, the PBAT fraction predominates in the B samples and PLA hardly crystallizes. The last statement would agree with the literature data that point out a very slow crystallization of PLA, especially under conditions of the actual experiment, when the cooling rate was 10 °C/min [50].

The cold crystallization of the PLA phase is still observed in the samples irradiated with the dose of at least 40 kGy. This is due to the reduction in the average molecular weight of the PBAT macromolecules that, because of essential degradation of them, may act as a nucleation agent and contribute to the cold crystallization of the PLA phase. At the same time, the two peaks assigned to the melting of the crystalline phase of PLA occur. The peak corresponding to the melting of the PBAT phase is invisible because, in the mentioned samples, the thermal effect of the cold crystallization of the PLA is larger than the thermal effect connected with the melting of the crystalline phase of PBAT, contrary to the effects observed in the samples irradiated with the smaller doses. The remaining effects, associated with the crystallization and melting of the B40 and B90 samples, are analogous to those of the L40 and L90 samples. Therefore, they are not discussed here.

## 4. Conclusions

The results of the investigation of the uncrosslinked fraction of the radiation modified PLA/PBAT blends discussed in the present article may contribute to a better understanding of some phenomena occurring during the radiation treatment of the biodegradable polymers and their blends. The applied research techniques enabled us to determine the most important changes occurring in the studied samples.

It was found that the electron radiation causes the occurrence of various effects in the biodegradable polymers and blends of them, which mostly depend on the composition of the materials being irradiated, radiation dose magnitude, and the presence of low molecular weight multifunctional compounds, such as TAIC. The investigation of the uncrosslinked fraction of the irradiated materials revealed an occurrence of effects, such as a different amount of that fraction, changes in the molecular weights of the macromolecules, the formation of new macromolecular structures, and thermal changes associated with, e.g., the cold crystallization or melting of crystalline phases. All the investigated samples contained the macromolecules of both PLA and PBAT, which indicated that any phase of both kinds of the blends was not completely crosslinked, independently of whether a given phase predominated or not. Much larger amounts of the uncrosslinked fractions occur in the samples of the predominating content of PBAT, which points out that this polymer is less susceptible than PLA to the radiation crosslinking in the presence of TAIC. This is mainly due to the presence in the PBAT structure of the aromatic groupings and the so-called protective effect. Samples with the predominating content of PLA more clearly exhibit the presence of the bimodal distribution of molecular weights and a larger reduction in the molecular weight magnitudes than the samples with the predominating content of PBAT. This shows that the intensity of the radiation degradation processes occurring in the studied blends increases as the weight fraction of PLA rises. The macromolecules present in the samples of both kinds of the uncrosslinked fractions exhibit different structures. In addition to the short macromolecules formed due to the radiation degradation, the short macromolecules being only an oligomeric fraction of the studied samples, which underwent neither crosslinking nor degradation, may be present. The longer macromolecules, in turn, are formed rather due to the lengthening by mutual binding at the chain ends or the formation of branched structures. There are no macromolecules crosslinked completely, which results from the occurrence of the cold crystallization and melting of the crystalline phases. The molecular weight of the macromolecules in the samples with the predominating content of PBAT decreases upon the irradiation more slowly compared to that in the samples with the predominating content of PLA. Therefore, the plasticization effect in the former samples is less evident than that in the latter samples. As a result, the glass transition temperature of the samples with the predominating content of PBAT decreases upon the irradiation more slowly than that of the samples with the predominating content of PLA. The shortening of the macromolecules in the PBAT phase, occurring with the increase in the electron radiation dose, may beneficially influence the process of the nucleation of the crystallization of the PLA phase, which usually is hindered and runs slowly. An increase in the electron radiation dose facilitates the recrystallization of the original crystalline structures into more stable forms, which is especially visible in the samples with the predominating content of PLA.

The results of the investigation of the uncrosslinked fraction of the radiation modified blends of PLA and PBAT presented in this article, constitute only a small fragment of wider considerations about the radiation treatment of the biodegradable polymers. Apart from that, there are many issues connected with the explanation and verification of some phenomena and hypotheses, which requires carrying out a larger number of additional studies. These concerns, e.g., the influence of the electron radiation on susceptibility to the biodegradation of the biodegradable polymers and their blends, the verification of the possibility of the occurrence of the specified mechanisms of crosslinking or degradation, and the specification of post-radiation effects. These issues should be the subjects of the next studies carried out by the scientists. Results presented in this article relate to issues of the radiation treatment of biodegradable materials. The modification of the properties of these materials by radiation treatment is poorly understood, and the modified materials in this way may be more widely used, e.g., in medicine, tissue engineering, or the packaging sector.

## Figures and Tables

**Table 1:** Symbols of the studied samples.

Composition	Dose (kGy)			
	0	10	40	90
PLA/PBAT 80/20 (L type samples)	L0	L10	L40	L90
PLA/PBAT 20/80 (B type samples)	B0	B10	B40	B90

**Table 2:** Number-average molecular weight (M_n_), weight-average molecular weight (M_w_), and polydispersion degree (PD) of the samples type L and B.

Sample	M_n_ (kDa)		M_w_ (kDa)		PD (M_w_/M_n_)	
	Peak 1	Peak 2	Peak 1	Peak 2	Peak1	Peak 2
L0	–	71.9	–	157.4	2.2	–
L10	–	51.4	–	159.9	3.1	–
L40	356.3	22.6	440.3	55.2	2.5	1.2
L90	253.0	13.0	354.4	33.3	2.6	1.4
B0	–	53.5	–	115.4	2.2	–
B10	–	50.3	–	158.9	3.2	–
B40	–	38.7	–	99.2	2.6	–
B90	393.2	21.2	458.5	50.3	2.4	1.2

**Table 3:** Data derived from the differential scanning calorimeter (DSC) curves recorded for the samples type L (Figure 6) and B (Figure 7).

Sample	T_g_ (°C)	T_cc_ (°C)	H_cc_ (J/g)	T_m_ (°C) (PBAT)	T_m_ (°C)(PLA)		H_m_(J/g) (PBAT)	H_m_*(J/g) (PLA)
					Peak 1	Peak 2		
L0	57.0	124.0	7.2	–	149.4	–	–	7.2
L10	54.6	120.7	12.9	–	147.5	153.3	–	13.0
L40	51.5	107.9	18.2	–	141.0	150.2	–	20.3
L90	48.2	102.2	17.5	–	136.3	147.1	–	21.6
B0	57.5	–	–	120.4	148.5	–	7.2	2.4
B10	56.2	–	–	120.2	148.3	–	8.8	0.8
B40	56.1	113.2	2.1	–	146.4	153.5	–	2.8
B90	54.1	103.9	4.2	–	142.2	150.5	–	4.2

* H_m_ = H_m, peak1_ + H_m, peak 2._
